# Non‐steroidal anti‐inflammatory drug use and inflammatory markers associated with gallbladder dysplasia: A case–control analysis within a series of patients undergoing cholecystectomy

**DOI:** 10.1002/ijc.35238

**Published:** 2024-10-31

**Authors:** Lorena Rosa, Paz Cook, Ruth M. Pfeiffer, Troy J. Kemp, Allan Hildesheim, Burcin Pehlivanoglu, Volkan Adsay, Enrique Bellolio, Juan Carlos Araya, Ligia Pinto, Catterina Ferreccio, Gloria Aguayo, Eduardo Viñuela, Jill Koshiol

**Affiliations:** ^1^ Facultad de Medicina Pontificia Universidad Católica de Chile Santiago Chile; ^2^ Advanced Center for Chronic Diseases Universidad de Chile and Pontificia Universidad Católica de Chile Santiago Chile; ^3^ Gillings School of Global Public Health University of North Carolina at Chapel Hill Chapel Hill North Carolina USA; ^4^ Infections and Immunoepidemiology Branch, Division of Cancer Epidemiology and Genetics National Cancer Institute Rockville Maryland USA; ^5^ Vaccine, Immunity and Cancer Directorate, Frederick National Laboratory for Cancer Research Frederick Maryland USA; ^6^ Department of Pathology Dokuz Eylul University Izmir Turkey; ^7^ Department of Pathology Koç University School of Medicine and Koç University Research Center for Translational Medicine Istanbul Turkey; ^8^ Departamento de Anatomía Patológica Universidad de La Frontera Temuco Chile; ^9^ Departamento de Patología Hospital Dr. Hernán Henríquez Aravena Temuco Chile; ^10^ Anatomía Patológica, Hospital Sótero del Río Santiago Chile; ^11^ UDA Hospital Sótero del Río, Facultad de Medicina Pontificia Universidad Católica de Chile Santiago Chile

**Keywords:** chemokines, cytokines, dysplasia, gallbladder cancer, inflammation, non‐steroidal anti‐inflammatory drugs

## Abstract

Inflammation has been associated with the development of gallbladder cancer (GBC). However, little is known about the associations of both, inflammation and the use of non‐steroidal anti‐inflammatory drugs (NSAIDs), with preneoplastic lesions. We analyzed the association of NSAIDs and gallbladder dysplasia in 82 patients with dysplasia and 1843 patients with gallstones among symptomatic patients from a high‐risk population. We also analyzed associations for 33 circulating immune‐related proteins in a subsample of all 68 dysplasia cases diagnosed at the time of sample selection and 136 gallstone controls. We calculated age‐ and sex‐adjusted odds ratios (ORs) and 95% confidence intervals (95% CIs). Biliary colic was reported among most cases (97.6%) and controls (83.9%). NSAID use was inversely associated with gallbladder dysplasia (OR: 0.48, 95%CI: 0.26–0.83). Comparing the highest versus lowest category of each immune‐related protein, eight proteins were inversely associated with dysplasia with sex‐ and age‐adjusted ORs ranging from 0.30 (95%CI: 0.12–0.77) for IL‐33 to 0.76 (95%CI: 0.59–0.99) for MIP‐1B. Of those, GRO remained associated with dysplasia (OR: 0.64, 95%CI: 0.45–0.91) and BCA‐1 was borderline associated (OR: 0.74, 95%CI: 0.54–1.01) after adjusting the logistic regression model for sex, age, and NSAIDs. In conclusion, NSAID users were less likely to have gallbladder dysplasia, suggesting that NSAIDs might be beneficial for symptomatic gallstones patients. The inverse association between immune‐related markers and dysplasia requires additional research, ideally in prospective studies with asymptomatic participants, to understand the role of the inflammatory response in the natural history of GBC and to address the biological effect of NSAIDs.

## Graphical abstract

What's new?

Gallbladder cancer is associated with inflammation, especially in the context of gallstones. However, little is known about the association between inflammation or the use of non‐steroidal anti‐inflammatory drugs and pre‐neoplastic lesions. In this case–control study of patients with symptomatic gallstones, non‐steroidal anti‐inflammatory drug use was inversely associated with gallbladder dysplasia. Moreover, circulating inflammatory markers tended to exhibit lower levels in patients with dysplasia compared to gallstone patients with less severe diagnoses. The identification of factors associated with dysplasia, the stage prior to gallbladder cancer, might help guide future prevention and early detection efforts.

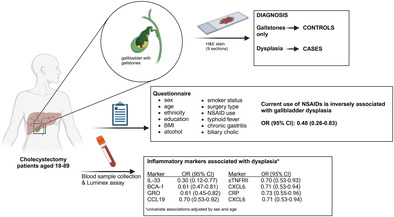

## 1 INTRODUCTION

Gallbladder cancer (GBC) is an aggressive cancer, with a median survival time of 6 months.^1^ Although GBC is rare worldwide, high incidence rates have been observed in South America, South Asia, and Southeast Asia.^2^ In particular, Chile has one of the highest age‐standardized incidence rates of 16/100,000 for women and 5/100,000 for men in the southern‐central region.^3^ The poor prognosis of GBC, mostly due to its late diagnosis and limited effective treatments, underlines the urgent need for early GBC detection and prevention strategies.

GBC is associated with inflammation, especially in the context of gallstones.^2^, ^4^, ^5^, ^6^, ^7^ Gallstones, which are the main risk factor for GBC, promote chronic inflammation due to persistent damage in the mucosa of the gallbladder.^4^, ^8^ The sustained inflammatory response may promote genetic alterations like *TP53* mutation and COX‐2 overexpression, contributing to the development of GBC through metaplasia‐dysplasia preneoplastic lesions.^4^ Histopathological changes in the gallbladder mucosa are associated with inflammation in patients with gallstones, characterized by high infiltration of neutrophils and macrophages, and upregulation of COX‐2.^5^, ^6^, ^7^ Additionally, we have shown that high levels of circulating inflammatory proteins were associated with gallstones and GBC.^9^, ^10^, ^11^ For example, gallstone patients had higher levels of IL‐6, IL‐10 and IL‐12p70 than population‐based controls,^9^ and GBC patients had increased levels of C‐reactive protein (CRP), CCL20, IL‐16, sTNFRI and sTNFRII compared to gallstones controls.^10^, ^11^ In the case of early stages of GBC, inflammation has been studied mainly in murine models. Increased infiltration of neutrophils and macrophages and upregulation of cytokines, such as IL‐1beta, have been shown in gallbladders from *in vivo* models of gallstones induced by a lithogenic diet.^8^, ^12^, ^13^ Moreover, patients with hyperplasia and metaplasia have been shown to have high infiltration of inflammatory cells in the gallbladder mucosa.^6^ However, the association of inflammatory markers and preneoplastic lesions in patients with gallstones is unclear.

In addition to gallstones, other risk factors like a high‐fat diet, obesity, and chronic bacterial infections, such as *Salmonella enterica* serovar Typhi, have also been associated with increased risk of GBC.^2^, ^14^, ^15^ These factors could contribute to a chronic inflammatory environment, promoting the development of GBC. In contrast, the use of non‐steroidal anti‐inflammatory drugs (NSAIDs) has been associated with decreased risk of GBC,^16^, ^17^, ^18^ as well as other inflammatory‐associated gastrointestinal cancers, such as colon and stomach cancer.^19^, ^20^ These studies suggest that inflammatory pathways could be targeted for cancer prevention. However, there are concerns about whether the observed associations are due to the biological effect of NSAIDs or bias, such as healthy‐user and healthy‐adherer effects. Hence, more evidence is needed to understand the potential protective effect of anti‐inflammatory drugs for GBC development.

To address these questions, we explored whether NSAID use impacts the gallbladder carcinogenesis process early by assessing the association of NSAIDs and inflammatory markers with gallbladder dysplasia, the precursor to GBC. Studying gallbladder dysplasia can provide valuable insight into how inflammation promotes GBC development and can provide more confidence in the etiologic factors associated with GBC. A better understanding of the factors associated with the stages of disease progression leading to GBC, including elucidation of risk factors for progression through the metaplasia‐dysplasia cascade, might help identify better strategies for cancer prevention and early detection of GBC.

## 2 METHODS

### 2.1 Study population and samples: the Cholecystectomy Research Study (CRS)

From May 2014 to May 2019, we enrolled 1949 cholecystectomy patients with ultrasound‐detected gallstones from three hospitals (Sótero del Río Hospital, Clínica Dávila and Public Hospital San José) in Santiago, Chile. These patients included all consecutive programmed cholecystectomy patients aged 18–89 years having surgery during business hours for whom we could collect pre‐surgery blood and a subset of patients having urgent surgery (on weekends or after hours) when staff were available for recruitment. Participants who were not mentally capable of answering the study questionnaire, had received a previous cholecystectomy, or for whom we could not obtain pre‐surgery blood were excluded from our study. Blood was collected from patients prior to cholecystectomy (before their histopathological diagnosis) to ensure that inflammatory proteins were not influenced by the surgery itself. Samples were processed within 3.5–5 h of blood draw and stored at −80°C for further analysis. Prior to surgery, we also collected epidemiologic information including sociodemographic and lifestyle data, and presence of biliary colic from each participant or their medical records. To increase the detection of gallbladder pre‐neoplastic lesions, for each patient, nine sections (3 sections from the neck, body, and fundus of the gallbladder) were processed and reviewed by hospital pathologists. For a subset of 117 participants (42 with diagnoses less severe than dysplasia, 62 low‐grade dysplasia, 3 unclassified dysplasia, 6 high‐grade dysplasia and 4 GBC), digital H&E slide images were reviewed by two independent‐blinded pathologists to confirm histopathological diagnosis; 96% of diagnoses agreed (112/117). For individuals with disagreement, a blinded third pathologist reviewed the digital images to determine the final diagnosis.

Of the 1949 CRS participants with gallstones, at the time of serum sample selection for immune markers measurement, 68 participants were diagnosed with gallbladder dysplasia (termed “dysplasia cases”; 60 low‐grade, 5 high‐grade and 3 indefinite dysplasia), 10 with GBC (termed “GBC cases”) and 1513 with less severe diagnoses (termed “controls”); the remaining 358 individuals received diagnoses after the time of sample selection (Figure S1). Controls were matched to dysplasia and GBC cases on sex, hospital and age (±5 years difference). From among all potential controls for each case, two were randomly selected (2:1 ratio) from those with a blood draw date no more than 60 days apart from the case. Thus, 68 dysplasia cases and 136 matched controls were analyzed to evaluate the associations of inflammatory proteins and dysplasia. Also, we explored the associations of these proteins and GBC using 10 GBC cases and 20 matched controls.

To study the associations of NSAIDs and dysplasia, we included the 14 dysplasia cases and 330 controls (344 total) who had not received diagnoses at the time of sample selection for the immune marker analysis (Figure S1). After confirmation of diagnosis, 1843 controls and 82 dysplasia cases (total *N* = 1925) were analyzed to evaluate the associations of NSAIDs and dysplasia. The 24 GBC cases were excluded from this analysis due to low sample size.

### 2.2 Laboratory procedures

We measured 66 circulating immune‐related proteins in 234 serum samples (68 dysplasia cases and 136 controls; 10 GBC cases and 20 controls) using the Milliplex assay (EMD Millipore, Billerica, MA, USA). Serum samples were incubated with beads in 96‐well plates and afterward, fluorescently labeled detection antibodies were added. The 96‐well plates were analyzed using a modified flow cytometer (Bio‐Plex 200, Bio‐Rad), and data were reported as pg/ml using Bio‐Plex Manager 6.1, as we have previously described.^10^, ^11^ To evaluate assay performance, we calculated the coefficients of variation (CVs) and intraclass correlation coefficients (ICCs) using 78 serum samples from 12 non‐CRS cholecystectomy subjects recruited in Temuco and Pucón, Chile.^21^ CVs and ICCs were calculated based on a linear regression model fit to the log‐transformed observed concentrations, as previously described.^9^ Analytes detectable in <25% of samples from these 12 non‐CRS subjects, with a CV > 30%, or an ICC < 0.7 were excluded from the final analysis. Thirty‐three of the 66 analytes (Table S1) were dropped due to these criteria, leaving 33 proteins to be analyzed.

### 2.3 Statistical analysis

Sociodemographic and lifestyle characteristics, including sex, age, self‐reported ethnicity (Chilean/Latino, Mapuche), education (0–8, 9–12 and ≥ 13 years of schooling), smoking status (never, former, current), body mass index (BMI; categorical: normal weight^1^: ≥18.5 to <25 kg/m^2^, overweight: ≥25 to <30 kg/m^2^ and obese: ≥30 kg/m^2^), consumption of alcohol during the last year (no alcohol and >1–30 days per month), surgery type (elective or urgent), current use of non‐aspirin NSAIDs (e.g., ibuprofen, ketorolac and diclofenac; use one or more times during the last week), and presence of biliary colic (ever/never; episode of severe pain in the pit of the stomach or under the right ribs that lasted more than 30 min without diarrhea), typhoid fever and chronic gastritis were compared between dysplasia cases and controls using chi‐square tests. We also explored the association of these variables with dysplasia using multivariable logistic regression models adjusted for sex and categorical age (<40, 40–50, 51–60 and > 60 years) in the minimally adjusted models and sex, age, the remaining sociodemographic and lifestyle variables, NSAID use, surgery type, biliary colic, typhoid fever and chronic gastritis in the fully adjusted models. Observations with missing data were excluded from the regression analyses. In addition, we assessed the association of NSAID use and dysplasia in normal weight, overweight and obese patients separately to identify effect modification of the NSAIDs‐dysplasia association, i.e. interactions of NSAID use and BMI. For this analysis, we fit logistic regression models within each BMI category, adjusting for sex and categorical age, and we evaluated the interaction between NSAIDs and BMI by adding an interaction term between NSAIDs and BMI to this model. Two‐sided Wald *p*‐values were calculated.

For the immune‐related protein analyses, as described previously,^10^, ^11^ samples with measurements below the lower limit of quantification (LLOQ) were assigned a value of half the LLOQ, and measurements above the upper limit of quantification (ULOQ) were assigned the ULOQ value. Immune‐related proteins were analyzed as categorical variables according to the proportion of subjects with detectable values as follows: (a) for proteins detectable in ≥75% of subjects, 4 categories were created based on quartiles of values above the LLOQ where subjects with undetectable values were included in the lowest quartile; (b) for detectable proteins in 50–75% of subjects, 4 categories were created: the first one included all subjects with undetectable values and the remaining 3 categories were based on tertiles of values above the LLOQ; (c) for detectable proteins in 25–50% of subjects, 3 categories were created: the first category included all subjects with undetectable values and the next 2 categories were split based on their median of the values above the LLOQ; (d) for low detectable proteins (<25%), 2 categories were created: one for values below the LLOQ and the other for values above the LLOQ.^10^, ^11^

For the biomarker component of the study, we first calculated partial Spearman rank correlations between the categorical immune‐related proteins and patient characteristics in cases and controls separately, controlling for categorical age and sex using the *pcor.test* function in the R package *ppcor*.^22^

To evaluate the association of circulating immune‐related proteins with dysplasia vs. controls, we included each marker separately in a conditional logistic regression model including categorical age (<40, 40–50, 51–60 and > 60 years) and sex. Immune‐related proteins were modeled as ordinal variables to evaluate linear trend, and *p*‐trend across categories was calculated using Wald tests. We provide the ordinal ORs, which reflect the linear change per category. We applied a multiple testing adjustment to all markers except for four a priori‐identified immune‐related markers, IL‐16, CCL20, sTNFRI and CRP, which increased the risk of early GBC (*p*‐values ≤ .001) in a prior study.^11^ Moreover, all immune‐related markers associated with dysplasia (*p*‐trend < .05) were included jointly in a multivariable logistic model, which was additionally adjusted for age, sex, and current use of NSAIDs. Furthermore, we fit a polytomous logistic regression model to assess the association of each marker, adjusted by categorical age and sex, with dysplasia and GBC outcomes as the case groups.

We explored the associations between current use of NSAIDs and each circulating immune‐related protein using ordinal logistic regression models with markers categories as the outcome variable and NSAID use as the independent predictor, adjusted for age and sex (minimally adjusted) and surgery type as additional adjustment (fully adjusted). For IL‐33, which had only two categories, we used a binomial logistic regression model. Characteristics of current NSAIDs users and non‐users were compared using chi‐square tests.

All statistical analyses were performed using R version 4.2.1.^23^

## 3 RESULTS

The 1925 CRS participants with and without dysplasia were similar except for current use of NSAIDs, biliary colic, and BMI (Table 1). Dysplasia cases were less likely to report current use of NSAIDs than controls (17.1% vs. 30.7%) and were more likely to report biliary colic (97.6% vs. 83.9%). Surprisingly, dysplasia cases were less likely to be obese than controls (25.6% vs. 38.7%).

**TABLE 1:** Characteristics of dysplasia cases and controls (*N* = 1925).

	Controls, *N* = 1843	Dysplasia, *N* = 82
Sex, N (%)		
Women	1441 (78.2)	64 (78.0)
Men	402 (21.8)	18 (22.0)
Age, mean ± SD (min–max)	45.8 ± 15.2 (18–89)	48.5 ± 15.0 (19–89)
Categorical age, *N* (%)		
< 40 years	690 (37.4)	21 (25.6)
40–50 years	440 (23.9)	28 (34.1)
51–60 years	364 (19.8)	17 (20.7)
> 60 years	349 (18.9)	16 (19.5)
Ethnicity, *N* (%)		
Chilean/Latino	1743 (94.6)	79 (96.3)
Mapuche	66 (3.6)	2 (2.4)
N/A	34 (1.8)	1 (1.2)
Education, *N* (%)		
0–8 years	515 (27.9)	22 (26.8)
9–12 years	928 (50.4)	43 (52.4)
≥ 13 years	284 (15.4)	10 (12.2)
N/A	116 (6.3)	7 (8.5)
Categorical BMI, *N* (%)		
Normal weight	349 (18.9)	25 (30.5)
Overweight	765 (41.5)	35 (42.7)
Obese	714 (38.7)	21 (25.6)
N/A	15 (0.8)	1 (1.2)
Consumption of alcohol in the last year, *N* (%)		
No alcohol	1168 (63.4)	54 (65.9)
More than 1 days per month	673 (36.5)	28 (34.1)
N/A	2 (0.1)	0 (0)
Smoker status, *N* (%)		
Never smoker	1579 (85.7)	75 (91.5)
Former smoker	104 (5.6)	3 (3.7)
Current smoker	160 (8.7)	4 (4.9)
Surgery type, *N* (%)		
Urgent	431 (23.4)	17 (20.7)
Elective	1411 (76.6)	65 (79.3)
N/A	1 (0.1)	0 (0)
Current use of NSAIDs, *N* (%)		
No	1277 (69.3)	68 (82.9)
Yes	565 (30.7)	14 (17.1)
N/A	1 (0.1)	0 (0)
Typhoid fever, *N* (%)		
No	1740 (94.4)	78 (95.1)
Yes	59 (3.2)	3 (3.7)
N/A	44 (2.4)	1 (1.2)
Chronic gastritis, *N* (%)		
No	1738 (94.3)	78 (95.1)
Yes	61 (3.3)	3 (3.7)
N/A	44 (2.4)	1 (1.2)
Biliary colic, *N* (%)		
No	258 (14.0)	2 (2.4)
Yes	1546 (83.9)	80 (97.6)
N/A	39 (2.1)	0 (0)

Abbreviation: N/A, not available.

Minimally and fully adjusted logistic models produced similar associations for dysplasia vs. controls (Table 2). In the fully adjusted model, NSAID use and obesity were inversely associated with dysplasia (OR: 0.37; 95% CI: 0.17–0.78 and OR: 0.42; 95% CI: 0.22–0.79, respectively). In contrast, biliary colic was positively associated with dysplasia (OR: 7.63; 95% CI: 2.31–47.13).

**TABLE 2:** Adjusted odds ratios (ORs) and 95% confidence intervals (95% CIs) for the association of patient characteristics and dysplasia (*N* = 1925).

Variable	Controls	Dysplasia	Minimally adjusted^a^		Fully adjusted^b^	
	*N* = 1843	*N* = 82	OR (95% CI)	*p*‐value	OR (95% CI)	*p*‐value
Ethnicity, *N* (%)						
Chilean/Latino	1743 (94.6)	79 (96.3)	Reference	—	Reference	—
Mapuche	66 (3.6)	2 (2.4)	0.69 (0.11–2.28)	.614	0.65 (0.10–2.19)	.561
Education, *N* (%)						
0–8 years	515 (27.9)	22 (26.8)	Reference	—	Reference	—
9–12 years	928 (50.4)	43 (52.4)	1.15 (0.65–2.07)		1.28 (0.71–2.37)	
≥ 13 years	284 (15.4)	10 (12.2)	0.96 (0.41–2.11)	.99	1.13 (0.47–2.56)	.652
Categorical BMI, *N* (%)						
Normal weight	349 (18.9)	25 (30.5)	Reference	—	Reference	—
Overweight	765 (41.5)	35 (42.7)	0.64 (0.38–1.10)		0.64 (0.36–1.13)	
Obese	714 (38.7)	21 (25.6)	0.41 (0.23–0.75)	.004	0.42 (0.22–0.79)	.007
Consumption of alcohol in the last year, *N* (%)						
No alcohol	1168 (63.4)	54 (65.9)	Reference	—	Reference	—
>1 days per month	673 (36.5)	28 (34.1)	0.91 (0.56–1.45)	.7	0.85 (0.50–1.40)	.530
Smoker status, N (%)						
Never smoker	1579 (85.7)	75 (91.5)	Reference	—	Reference	—
Former smoker	104 (5.6)	3 (3.7)	0.64 (0.15–1.78)		0.81 (0.19–2.36)	
Current smoker	160 (8.7)	4 (4.9)	0.57 (0.17–1.39)	.207	0.49 (0.12–1.37)	.227
Surgery type, *N* (%)						
Urgent	431 (23.4)	17 (20.7)	Reference	—	Reference	—
Elective	1411 (76.6)	65 (79.3)	1.11 (0.66–1.98)	.714	0.72 (0.35–1.58)	.4
Current use of NSAIDs, *N* (%)						
No	1277 (69.3)	68 (82.9)	Reference	—	Reference	—
Yes	565 (30.7)	14 (17.1)	0.48 (0.26–0.83)	.014	0.37 (0.17–0.78)	.012
Typhoid fever, *N* (%)						
No	1740 (94.4)	78 (95.1)	Reference	—	Reference	—
Yes	59 (3.2)	3 (3.7)	1 (0.24–2.80)	1	1.25 (0.29–3.66)	.716
Chronic gastritis, *N* (%)						
No	1738 (94.3)	78 (95.1)	Reference	—	Reference	—
Yes	61 (3.3)	3 (3.7)	1.04 (0.25–2.89)	.954	0.95 (0.22–2.71)	.927
Biliary colic, *N* (%)						
No	258 (14.0)	2 (2.4)	Reference	—	Reference	—
Yes	1546 (83.9)	80 (97.6)	7.28 (2.26–44.52)	.006	7.63 (2.31–47.13)	.005

*Note*: *p*‐value was calculated by Wald test.

^a^ Adjusted for categorical age (<40, 40–50, 51–60 and > 60 years) and sex.

^b^ Adjusted for categorical age, sex, Mapuche ethnicity, education, smoker habit, alcohol consumption, current use of NSAIDs, surgery type, typhoid fever, chronic gastritis and biliary colic.

We further evaluated a possible interaction of obesity with NSAIDs given that obesity is also related to inflammation^24^ and GBC^15^ by first evaluating the association of NSAID use and dysplasia within each BMI category (Table S2). We found that NSAID users were less likely to have dysplasia with an OR of 0.30 (95% CI: 0.07–0.90) compared to non‐users among patients with normal weight. The association was weaker among overweight (OR: 0.49, 95% CI: 0.18–1.12) and obese (OR: 0.70, 95% CI: 0.23–1.84) participants. However, there was no evidence of interaction on a multiplicative scale between categorical BMI and current use of NSAIDs (*p*_interaction_ of .5 and .2 for the interaction term of overweight and obesity with NSAID use; respectively), although sample size was limited.

### 3.1 Association of circulating inflammatory markers and dysplasia

**FIGURE 1:**
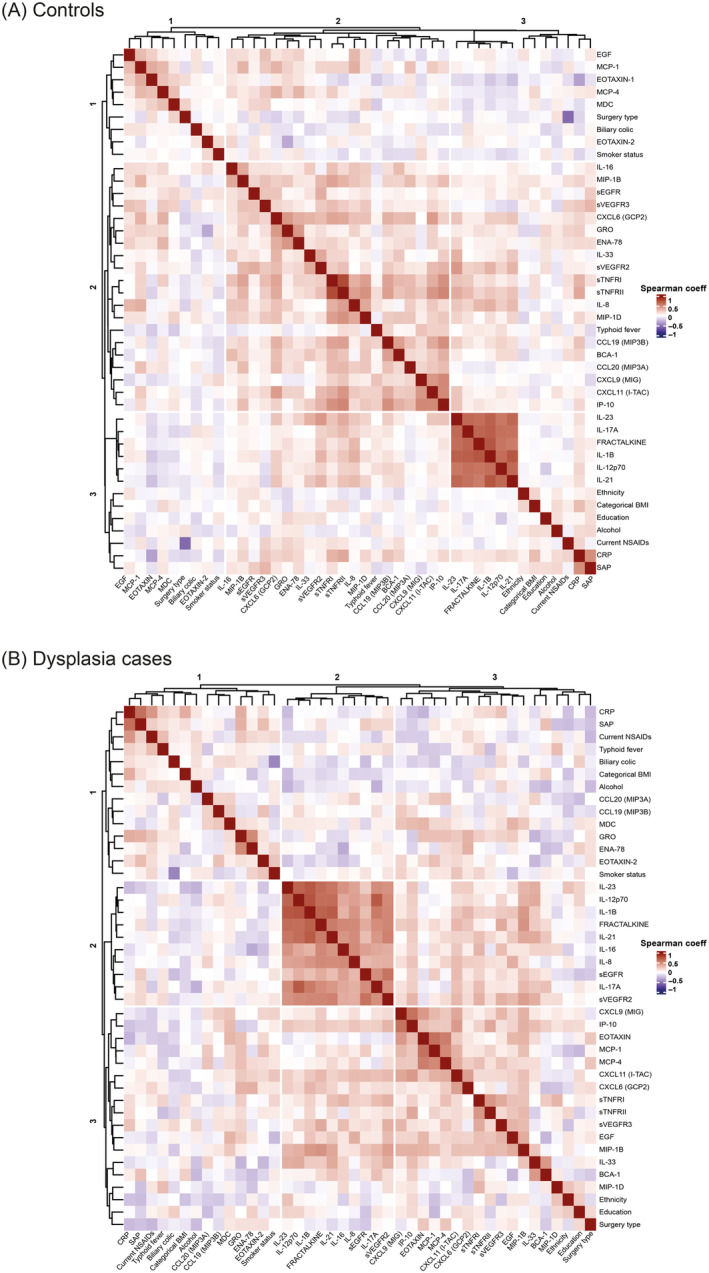
Spearman rank correlations adjusted for categorical age and sex of inflammatory markers and patient characteristics in controls and dysplasia cases. (A) Controls and (B) Dysplasia cases.

In a subset of 204 participants (68 dysplasia cases and 136 matched‐controls), we evaluated the association between circulating inflammatory markers and dysplasia. First, we explored the correlations between inflammatory markers in controls and dysplasia cases, separately. We identified two groups of strongly positively correlated (coeff ≥ 0.6) immune‐related markers in both, controls and cases. The first group included IL‐23, FRACTALKINE, IL‐17A, IL‐12p70, IL‐1B and IL‐21, and the second group CXCL9, IP‐10, sTNFRI, sTNFRII (Figure 1A,B; Table S3).

**FIGURE 2:**
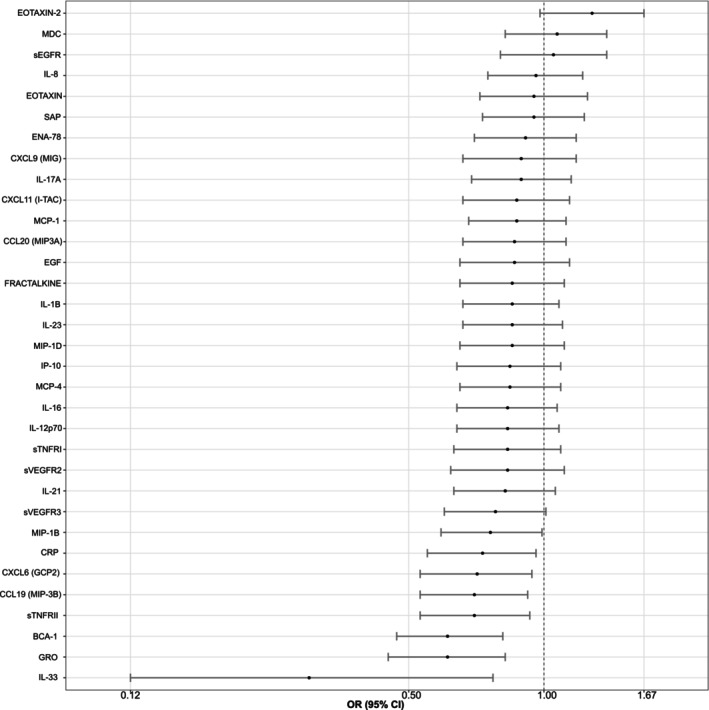
Associations of inflammatory markers and dysplasia. All binomial logistic regression models were adjusted by categorical age and sex. Odds ratios (ORs) and 95% confidence intervals (95% CIs) are shown on the logarithmic scale. All markers had 4 categories except IL‐33 which had 2 categories. Only BCA‐1 and GRO passed Bonferroni correction (*p*‐value < .0015).

Next, we evaluated associations between the markers and dysplasia. Eight of the 33 immune‐related markers (IL‐33, BCA‐1, GRO, CCL19 (MIP‐3B), sTNFRII, CXCL6 (GCP2), CRP and MIP‐1B) were inversely associated with dysplasia, with univariate ORs ranging from 0.30 (95%CI: 0.12–0.77) for IL‐33 to 0.76 (95%CI: 0.59–0.99) for MIP‐1B (Figure 2; Table S4). After Bonferroni adjustment, only GRO and BCA‐1 remained statistically significantly associated (Table S4). The eight markers that exhibited univariate associations were not strongly correlated with each other (Spearman coeff < 0.6; Table S3). In a logistic regression model that included these eight markers jointly (adjusted for age, sex, and current use of NSAIDs), only GRO remained statistically significantly inversely associated with dysplasia with an OR (95% CI) of 0.64 (0.45–0.91), although BCA‐1 showed a borderline association with dysplasia (OR: 0.74, 95%CI: 0.54–1.01; Table S4).

Further, we explored the associations between inflammatory markers and GBC. More markers tended to be positively associated with GBC versus controls than dysplasia versus controls (Figure S2). For example, the OR for the association between CRP and GBC was 1.69 (95% CI: 0.86–3.34), whereas the association with dysplasia was inverse (OR: 0.71, 95% CI: 0.54–0.95) (Figure S2; Table S4). However, the number of GBC cases was very small (*N* = 10), requiring a larger study to fully characterize these associations.

### 3.2 Circulating inflammatory markers in NSAIDs users

To illuminate the inverse association between immune‐related markers and dysplasia, we evaluated the associations between current use of NSAIDs and immune‐related markers in controls and dysplasia cases, separately. After adjusting for categorical age, sex and surgery type, current use of NSAIDs was associated with increased levels of sVEGFR3 (OR: 2.61, 95% CI: 1.12–6.33) and GRO (OR: 2.55, 95% CI: 1.11–6.01) in controls and with higher levels of CRP (OR: 4.61, 95% CI: 1.47–15.64) in dysplasia cases (Tables 3 and 4).

**TABLE 3:** Adjusted odds ratios (ORs) and 95% confidence intervals (95% CIs) from ordinal logistic regression models for the association of current use of NSAIDs and each inflammatory marker among gallstones controls.

Outcome: categorical marker	Minimally adjusted^a^		Fully adjusted^b^	
	OR (95% CI)	*p*‐value	OR (95% CI)	*p*‐value
sVEGFR3	2.53 (1.26–5.19)	.010	2.61 (1.12–6.33)	.030
GRO	2.30 (1.13–4.76)	.023	2.55 (1.11–6.01)	.029
CRP	2.11 (1.07–4.17)	.031	1.99 (0.88–4.55)	.101
ENA‐78	1.94 (0.98–3.88)	.059	1.65 (0.74–3.72)	.223
BCA‐1	1.87 (0.95–3.72)	.071	2.62 (1.16–6.05)	.022
MIP‐1B	1.80 (0.92–3.57)	.089	1.63 (0.74–3.60)	.221
sTNFRII	1.79 (0.87–3.70)	.112	1.81 (0.79–4.13)	.159
sTNFRI	1.62 (0.82–3.19)	.164	1.53 (0.70–3.38)	.289
MCP‐1	1.57 (0.81–3.07)	.179	1.38 (0.63–3.04)	.416
CXCL6 (GCP2)	1.55 (0.80–3.05)	.198	1.11 (0.50–2.45)	.804
CCL19 (MIP‐3B)	1.54 (0.79–3.04)	.208	2.46 (1.09–5.70)	.032
IL‐21	1.41 (0.72–2.75)	.313	1.09 (0.49–2.41)	.836
sVEGFR2	1.38 (0.71–2.69)	.349	1.99 (0.89–4.51)	.094
MCP‐4	1.25 (0.64–2.41)	.514	1.15 (0.52–2.51)	.725
IL‐8	1.21 (0.62–2.39)	.573	0.78 (0.35–1.72)	.539
IL‐16	1.17 (0.58–2.35)	.653	1.35 (0.58–3.12)	.485
MIP‐1D	1.11 (0.55–2.23)	.777	1.46 (0.65–3.32)	.360
CXCL11 (I‐TAC)	1.09 (0.56–2.14)	.799	1.07 (0.49–2.34)	.865
CXCL9 (MIG)	1.08 (0.55–2.11)	.832	1.18 (0.54–2.60)	.679
EOTAXIN‐2	1.08 (0.54–2.14)	.834	0.95 (0.43–2.10)	.901
IL‐17A	1.03 (0.53–1.99)	.934	0.71 (0.33–1.54)	.393
CCL20 (MIP‐3A)	0.99 (0.50–1.92)	.966	1.04 (0.46–2.37)	.920
IL‐12p70	0.99 (0.51–1.93)	.988	0.84 (0.37–1.89)	.676
IL‐1B	0.94 (0.48–1.84)	.85	0.87 (0.39–1.93)	.738
IL‐23	0.90 (0.46–1.76)	.749	0.90 (0.40–2.00)	.796
SAP	0.88 (0.45–1.71)	.707	0.84 (0.38–1.84)	.663
sEGFR	0.85 (0.43–1.67)	.636	1.04 (0.47–2.29)	.931
MDC	0.82 (0.41–1.60)	.552	1.47 (0.65–3.36)	.353
EGF	0.74 (0.37–1.47)	.396	0.62 (0.27–1.40)	.252
IP‐10	0.71 (0.35–1.41)	.326	0.82 (0.36–1.86)	.640
FRACTALKINE	0.63 (0.32–1.26)	.196	0.57 (0.25–1.26)	.165
EOTAXIN	0.59 (0.30–1.15)	.12	0.72 (0.31–1.65)	.432

^a^ Adjusted for categorical age and sex.

^b^ Adjusted for categorical age, sex and elective versus urgent surgery.

**TABLE 4:** Adjusted odds ratios (ORs) and 95% confidence intervals (95% CIs) from ordinal logistic regression models for the association of current use of NSAIDs and each inflammatory marker among dysplasia cases.

Outcome: categorical marker	Minimally adjusted^a^		Fully adjusted^b^	
	OR (95% CI)	*p*‐value	OR (95% CI)	*p*‐value
CRP	5.70 (1.83–19.27)	.004	4.61 (1.47–15.64)	.011
SAP	2.47 (0.81–7.83)	.116	2.09 (0.67–6.71)	.207
GRO	2.38 (0.80–7.22)	.120	2.71 (0.88–8.59)	.084
EOTAXIN‐2	1.77 (0.58–5.61)	.317	2.07 (0.67–6.68)	.209
CXCL6 (GCP2)	1.71 (0.57–5.27)	.341	2.12 (0.67–6.85)	.201
MIP‐1D	1.60 (0.50–5.11)	.425	1.60 (0.48–5.23)	.436
ENA‐78	1.59 (0.55–4.70)	.397	1.72 (0.56–5.37)	.341
MCP‐4	1.52 (0.49–4.73)	.463	1.70 (0.54–5.43)	.364
sTNFRI	1.26 (0.42–3.76)	.677	1.24 (0.39–3.89)	.711
sVEGFR2	1.21 (0.39–3.80)	.743	1.18 (0.36–3.89)	.788
IL‐21	1.13 (0.36–3.50)	.827	0.98 (0.30–3.12)	.966
CCL20 (MIP‐3A)	1.12 (0.30–3.97)	.861	1.12 (0.29–4.11)	.870
BCA‐1	1.09 (0.35–3.29)	.880	0.86 (0.26–2.69)	.798
MCP‐1	1.05 (0.36–3.02)	.927	1.01 (0.34–2.96)	.987
CXCL11 (I‐TAC)	1.01 (0.33–3.10)	.980	0.93 (0.30–2.93)	.907
sTNFRII	0.93 (0.29–2.95)	.902	0.76 (0.22–2.53)	.660
IL‐16	0.87 (0.26–2.77)	.821	0.76 (0.21–2.60)	.668
IL‐8	0.78 (0.22–2.71)	.701	0.63 (0.16–2.27)	.480
MIP‐1B	0.71 (0.21–2.31)	.575	0.68 (0.20–2.25)	.527
MDC	0.63 (0.19–1.99)	.429	0.68 (0.20–2.28)	.532
sEGFR	0.62 (0.19–2.02)	.430	0.47 (0.14–1.58)	.223
sVEGFR3	0.61 (0.18–1.99)	.412	0.60 (0.17–2.03)	.408
IL‐12p70	0.60 (0.18–1.90)	.391	0.57 (0.17–1.85)	.356
CCL19 (MIP‐3B)	0.59 (0.18–1.83)	.367	0.45 (0.13–1.47)	.197
IL‐1B	0.52 (0.13–1.84)	.322	0.44 (0.11–1.60)	.225
IL‐23	0.50 (0.15–1.67)	.266	0.47 (0.13–1.60)	.232
EOTAXIN	0.49 (0.14–1.61)	.241	0.59 (0.17–2.08)	.412
FRACTALKINE	0.47 (0.14–1.55)	.222	0.45 (0.13–1.49)	.193
IP‐10	0.47 (0.14–1.51)	.209	0.41 (0.12–1.35)	.147
IL‐17A	0.42 (0.12–1.32)	.147	0.41 (0.12–1.32)	.145
EGF	0.38 (0.11–1.32)	.129	0.37 (0.10–1.34)	.132
CXCL9 (MIG)	0.37 (0.09–1.33)	.138	0.29 (0.07–1.09)	.076

^a^ Adjusted for categorical age and sex.

^b^ Adjusted for categorical age, sex and elective versus urgent surgery.

Given the positive association of NSAIDs with sVEGFR3 and GRO in gallstones controls and with CRP in dysplasia cases, we added these three markers to the logistic regression model adjusted for age, sex and biliary colic for the subset of people with immune‐related marker data. Adding these three markers to the model attenuated the inverse association between NSAIDs and dysplasia from 0.57 (95% CI: 0.27–1.14) to 0.79 (95% CI: 0.36–1.68).

Further, we compared the characteristics of current NSAID users to non‐current users among all 1925 CRS patients (for controls and dysplasia cases, separately). Notably, among controls, current NSAID users were more likely to undergo urgent surgery (65.8% vs. 4.6%) and to report biliary colic (95.2% vs. 78.9%) than non‐current NSAID users (Table S5). Similarly, among dysplasia cases, urgent surgery was more common in NSAID users than non‐users (50% vs. 14.7%, Table S6).

## 4 DISCUSSION

To our knowledge, this is the first case–control study to evaluate the associations of NSAID use and circulating immune‐related markers with gallbladder dysplasia among patients with symptomatic gallstones. We found that patients with dysplasia had lower levels of IL‐33, BCA‐1, GRO, CCL19 (MIP‐3B), sTNFRII, CXCL6 (GCP2), CRP and MIP‐1B compared to gallstones patients without dysplasia. When evaluating all CRS participants, we found that NSAID use was inversely associated with gallbladder dysplasia, with an OR of 0.37 (95% CI: 0.17–0.78).

An important aspect of our study is that all CRS patients have gallstones, which promote inflammation.^2^, ^25^ While the majority of individuals with gallstones are asymptomatic,^25^ most CRS patients (cases and controls) experienced biliary colic. Some may have taken NSAIDs to alleviate their symptoms. However, if the association were entirely due to biliary colic, one would expect to see a positive association between NSAID use and dysplasia. In our study, NSAIDs remained inversely associated with dysplasia after biliary colic adjustment, suggesting that the inverse association of NSAIDs and dysplasia was not biased due to symptoms.

The association of NSAIDs and dysplasia might also be influenced by other inflammation‐related factors, like obesity.^24^ Obese patients might have increased levels of inflammatory proteins compared with non‐obese patients. Hence, the use of NSAIDs might not have the same effect among all CRS patients. While the interaction between categorical BMI and NSAIDs was not statistically significant, the inverse association with dysplasia was strongest among patients with normal weight and weakest among obese patients. Similarly, Wang et al. found that NSAID use was strongly associated with reduced CRC risk among patients with normal weight whereas a mild protective effect was observed among overweight and obese patients.^26^ Taken together, these findings suggest that individuals with normal weight might be more likely to benefit from NSAID use.

Another concern might be that among symptomatic gallstone patients, those with severe symptoms might be more likely to use NSAIDs and undergo emergency surgery, potentially going to surgery before they develop dysplasia. We found that NSAID users were more likely to undergo urgent surgery. Hence, the inverse association of NSAIDs and dysplasia might be explained in part to earlier surgery of those patients with severe symptoms. While this explanation seems less likely given the strong association between biliary colic and dysplasia (OR: 7.63; 95% CI: 2.31–47.13), it is possible that symptom severity could have an impact. To avoid these issues, the association of NSAIDs and dysplasia would ideally be evaluated in ultrasound‐detected gallstones patients, regardless of whether the gallstones are symptomatic or not. For example, the prospective Chile Biliary Longitudinal Study (Chile BiLS)^27^ offers a valuable setting for studying the potential impact of NSAIDs on gallbladder preneoplasia and will provide significant insights for a better understanding of the natural history of GBC. In particular, longitudinal measurements of immune‐related markers are crucial to evaluate the potential beneficial effect of NSAID use on dysplasia and GBC.

This study also provides some hints as to a potential biologic mechanism for the inverse association between NSAID use and dysplasia. In the subset of participants with immune‐related marker measurements, the age‐, sex‐ and biliary colic‐adjusted association between current use of NSAIDs and dysplasia was inverse, although not statistically significant (OR: 0.57, 95% CI: 0.27–1.14). After including sVEGRF3, GRO and CRP (the immune‐related markers associated with current use of NSAIDS) in the model, the association between NSAID use and dysplasia was attenuated (OR: 0.79, 95% CI: 0.36–1.68). While this analysis was limited by sample size, the findings support the hypothesis that the association between NSAIDs and dysplasia might be mediated through immune‐related processes.

Given that all participants in our study had gallstones, which cause tissue injury, promoting inflammation,^2^, ^25^ we expected the use of NSAIDs to reduce the levels of inflammation in our participants. Contrary to our expectations, current use of NSAIDs was associated with increased levels of sVEGFR3 and GRO in controls and with high levels of CRP in dysplasia cases after adjustment for age, sex, and surgery type. We also observed a higher frequency of biliary colic in NSAID users compared to non‐users, which could be related to having increased inflammation due to more acute changes in the gallbladder in NSAID users. Greater frequency of acute gallbladder changes could also explain the higher likelihood of urgent (emergency) surgeries among NSAID users compared to non‐users. Consistent with our findings, one study of colorectal adenoma reported that the use of NSAIDs was positively associated with high levels of other inflammatory proteins, IL‐6 and TNF‐alpha, which the authors concluded could be due to confounding by indication.^28^ Taken together, these results suggest that NSAID users could have higher baseline levels of immune‐related proteins due to having more symptoms and acute changes in the gallbladder than non‐users, resulting in a positive association between NSAIDs and inflammatory markers. For an accurate assessment of the effect of NSAIDs on systemic inflammation, longitudinal studies of immune‐related proteins levels before and after taking NSAIDs are warranted.

Our results could also be influenced by the duration of NSAID use, which was not available in our study. Short‐ and long‐term of NSAIDs has been associated with a reduced risk of gastrointestinal cancers. However, the strength of the effect seems to vary depending on the duration of NSAID use. In a randomized trial of 1121 patients with colorectal adenomas, one‐year of low‐dose of aspirin (81 mg per day) showed a moderate benefit in patients with advanced adenomas compared to the placebo group, with a relative risk of 0.59 (95% CI: 0.38–0.92).^29^ Additionally, a mild effect of a higher doses of aspirin (160 or 300 mg/day) over 1 year, showed a relative risk of 0.73 (95% CI: 0.52–1.04) in patients with a history of colorectal adenomas in the APACC intervention Trial.^30^ However, a prospective cohort study of 82,911 women showed that regular aspirin use ≥2 standard [325‐mg] tablets within 1–5 and 6–10 years was not significantly associated with reduced risk of colorectal cancer. A significant reduction in colorectal cancer risk was only shown in women who regularly used aspirin for more than 10 years of use (*p*_trend_ ≤ .001).^31^

Short‐term use of NSAIDs may not be sufficient to reduce the high levels of inflammation seen in these symptomatic gallstones patients, resulting in elevated inflammatory markers in current‐users compared to non‐users, and/or in a small inverse association with gallbladder dysplasia. Measurement of the duration of NSAID use would facilitate understanding of potential differences in the effect of short‐ and long‐term NSAID use on disease association. Even without information on duration of use, however, we showed an inverse association of NSAIDs with gallbladder dysplasia (OR: 0.48, 95%CI: 0.26–0.83).

We were also surprised to see a trend toward inverse associations between immune‐related markers and gallbladder dysplasia given that gallstone‐related GBC is associated with chronic inflammation^4^, ^8^, ^10^, ^11^, ^32^ and higher levels of immune‐related markers compared to gallstone controls.^10^, ^11^ However, our inverse associations with dysplasia are consistent with a previous study that found that IL‐2, IL‐5, IL‐8, IL‐10, IL‐13 and TNF‐alpha tend to be downregulated in low‐grade/moderate dysplasia of the pancreas compared to serous cystadenoma without dysplasia.^33^ Interestingly, in our study, the majority of dysplasias were low‐grade. In the case of advanced lesions, pro‐inflammatory cytokines like L‐1beta, IL‐5, IL‐6, IL‐8 and TNF‐alpha were elevated in high‐grade dysplasia/pancreatic cancer compared to low‐grade/moderate dysplasia group,^33^, ^34^ suggesting that immune response patterns may change across the natural history of disease.

Furthermore, Delgiorno et al. and Leary et al. have shown that the levels of inflammation can be regulated by the presence of tuft cells, which are commonly present in the normal digestive and biliary tracts, and the amount of tuft cells can change as cancer progresses.^35^, ^36^, ^37^ Delgiorno et al. (2014) found that these cells are present in higher amounts in metaplasia but absent in pancreatic cancer. In addition, they showed that the depletion of these cells accelerates tumor progression in vivo accompanied by upregulation of pro‐inflammatory cytokines.^36^ Furthermore, Leary et al.^37^ showed that that the depletion of these cells in the gallbladder promotes neutrophil infiltration and upregulation of cytokines, which could favor tumor progression. Moreover, gallstone formation could be promoted by tuft cells due to their sensitivity to bile acids, which can favor the development of GBC.^37^ Taken together, these findings suggest that levels of inflammatory proteins might change across metaplasia‐dysplasia‐GBC sequence which might explained the inverse association between inflammatory markers and dysplasia. Longitudinal studies are warranted to study the associations of inflammatory markers and gallbladder preneoplastic lesions.

Although we previously observed higher levels of immune‐related markers in GBC cases,^10^, ^11^ we had limited ability to evaluate associations with GBC in the current study given the small number of cases. However, the association with inflammatory markers tended to be in a positive direction more often for GBC than for dysplasia in the current study. Longitudinal studies with larger numbers of GBC and dysplasia cases are warranted to assess inflammatory markers in the progression of GBC.

This study has several strengths. It is the first to our knowledge to assess the association of NSAID use and inflammatory markers with dysplasia, a precursor to GBC. We included surgery type and biliary colic in our analysis to address confounding by indication. Diagnosis misclassification was minimized through an extensive pathology review. Our study also has some limitations. For example, the size of the subsample with immune‐related marker data limited the power to assess complex associations. Larger sample sizes are also needed to address the association between inflammation and GBC. Although we evaluated confounders, our results could be influenced by additional patient characteristics such as diet, use of medications and exercise, which were not measured in our study.

The potential for confounding is always of concern in studies of NSAIDs. Although symptoms can influence NSAID use, the inverse association between NSAID use and dysplasia remained after adjusting for symptoms, including biliary colic, and multiple other confounders. Diet, which was not measured in our study, could also affect the association of NSAIDs and gallbladder dysplasia since symptomatic patients may avoid high‐fat diets to prevent nausea and bloating, cause for example by fried and fatty foods,^25^ and be more likely to use NSAIDs to alleviate pain from gastrointestinal symptoms. While we did not have information on diet, we did adjust for obesity, which is correlated with dietary behaviors. Taken together, we think it likely that the inverse association between NSAIDs and gallbladder dysplasia reflects a true association.

In summary, we found that NSAID use was inversely associated with gallbladder dysplasia among symptomatic patients. Additional studies, especially longitudinal studies with rich biorepositories, are needed to better understand whether NSAID use is biologically associated with gallbladder dysplasia and cancer, and if so, how NSAIDs influence disease progression and might be leveraged in effective prevention strategies. Moreover, immune‐related markers tended to be inversely associated with dysplasia, suggesting a regulation of immune response at early stages in the natural history of GBC. Future studies at early stages of precursor lesions are warranted to understand the role of chronic inflammation and immune response in GBC progression.

## AUTHOR CONTRIBUTIONS

**Lorena Rosa:** Data curation; formal analysis; investigation; methodology; visualization; writing – original draft; writing – review and editing. **Paz Cook:** Conceptualization; formal analysis; investigation; methodology; writing – review and editing. **Ruth M. Pfeiffer:** Conceptualization; formal analysis; investigation; methodology; supervision; writing – review and editing. **Troy J. Kemp:** Investigation; writing – review and editing. **Allan Hildesheim:** Conceptualization; formal analysis; writing – review and editing. **Burcin Pehlivanoglu:** Investigation; writing – review and editing. **Volkan Adsay:** Investigation; writing – review and editing. **Enrique Bellolio:** Investigation; writing – review and editing. **Juan Carlos Araya:** Investigation; writing – review and editing. **Ligia Pinto:** Investigation; writing – review and editing. **Catterina Ferreccio:** Conceptualization; formal analysis; funding acquisition; methodology; writing – review and editing. **Gloria Aguayo:** Conceptualization; investigation; methodology; writing – review and editing. **Eduardo Viñuela:** Conceptualization; investigation; methodology; writing – review and editing. **Jill Koshiol:** Conceptualization; formal analysis; funding acquisition; investigation; methodology; supervision; writing – original draft; writing – review and editing.

## FUNDING INFORMATION

This work was supported by general funds from the Intramural Research Program of the US National Institutes of Health, National Cancer Institute, Division of Cancer Epidemiology and Genetics, and the Office of Research on Women's Health, National Institutes of Health. This research was also funded by Fondo Nacional de Desarrollo Científico y Tecnológico (FONDECYT Regular 1,212,066) and Fondo de Financiamiento de Centros de Investigación en Áreas Prioritarias (FONDAP) (grant number 15130011).

## CONFLICT OF INTEREST STATEMENT

None declared.

## ETHICS STATEMENT

All participants provided written consent for biospecimen and questionnaire data collection, and the Pontificia Universidad Católica de Chile and the Chilean Ministry of Health ethical review boards approved this study.

## PREVIOUS PRESENTATION

This work was previously presented in the poster section at the Annual Meeting of the American Association for Cancer Research (AACR) in San Diego, California, USA, and was published as part of the conference proceedings in the abstract book: *Cancer Res* (2024) 84 (6_Supplement): 3446.

## Supporting information


Appendix S1.

## Data Availability

The data contributing to the paper will be deposited in the Division of Cancer Epidemiology and Genetics (DCEG‐PDR) at the National Cancer Institute, under controlled access with the following data use limitations: disease‐specific (gallbladder disease), sensitive data, such as Mapuche ethnicity, will not be shared. Further information is available from the corresponding authors upon request.

## Endnote

^1^
Only 4 of 1925 participants (0.2%) had BMI lower than 18.5 kg/m^2^. Given that BMI values 17.6–18.4 kg/m^2^ were rare in our study, we did not consider underweight as a separate category but rather included these participants in the normal weight category. There was no substantive difference in the results if these individuals were excluded.

## References

[1] Hundal R , Shaffer E . Gallbladder cancer: epidemiology and outcome. Clin Epidemiol. 2014;6:99‐109. 24634588 10.2147/CLEP.S37357 PMC3952897

[2] Roa JC , García P , Kapoor VK , Maithel SK , Javle M , Koshiol J . Gallbladder cancer. Nat Rev Dis Primers. 2022;8(1):69. 36302789 10.1038/s41572-022-00398-y PMC12314663

[3] Miranda‐Filho A , Piñeros M , Ferreccio C , et al. Gallbladder and extrahepatic bile duct cancers in the Americas: incidence and mortality patterns and trends. Int J Cancer. 2020;147(4):978‐989. 31922259 10.1002/ijc.32863 PMC8629410

[4] Espinoza JA , Bizama C , García P , et al. The inflammatory inception of gallbladder cancer. Biochimica et Biophysica Acta. 2016;1865(2):245‐254. 26980625 10.1016/j.bbcan.2016.03.004 PMC6287912

[5] Carotti S , Guarino MPL , Cicala M , et al. Effect of ursodeoxycholic acid on inflammatory infiltrate in gallbladder muscle of cholesterol gallstone patients. Neurogastroenterol Motility. 2010;22(8):866. 10.1111/j.1365-2982.2010.01510.x 20426797

[6] Roa I , De Aretxabala X , Araya JC , Roa J . Preneoplastic lesions in gallbladder cancer. J Surg Oncol. 2006;93:615‐623. 16724345 10.1002/jso.20527

[7] Kanoh K , Shimura T , Tsutsumi S , et al. Significance of contracted cholecystitis lesions as high risk for gallbladder carcinogenesis. Cancer Lett. 2001;169(1):7‐14. 11410319 10.1016/s0304-3835(01)00523-7

[8] Rosa L , Lobos‐González L , Muñoz‐Durango N , et al. Evaluation of the chemopreventive potentials of ezetimibe and aspirin in a novel mouse model of gallbladder preneoplasia. Mol Oncol. 2020;14(11):2834‐2852. 33326125 10.1002/1878-0261.12766 PMC7607176

[9] Liu Z , Kemp TJ , Gao YT , et al. Association of circulating inflammation proteins and gallstone disease. J Gastroenterol Hepatol. 2018;33(11):1920‐1924. 29671891 10.1111/jgh.14265 PMC7576672

[10] Koshiol J , Castro F , Kemp TJ , et al. Association of inflammatory and other immune markers with gallbladder cancer: results from two independent case‐control studies. Cytokine. 2016;83:217‐225. 27173614 10.1016/j.cyto.2016.05.003 PMC4876019

[11] Koshiol J , Gao YT , Corbel A , et al. Circulating inflammatory proteins and gallbladder cancer: potential for risk stratification to improve prioritization for cholecystectomy in high‐risk regions. Cancer Epidemiol. 2018;54:25‐30. 29554539 10.1016/j.canep.2018.03.004 PMC5971138

[12] Van Erpecum KJ , Wang DQH , Moschetta A , et al. Gallbladder histopathology during murine gallstone formation: relation to motility and concentrating function. J Lipid Res. 2006;47(1):32‐41. 16224116 10.1194/jlr.M500180-JLR200

[13] Rege RV , Prystowsky JB . Inflammation and a thickened mucus layer in mice with cholesterol gallstones. J Surg Res. 1998;74(1):81‐85. 9536979 10.1006/jsre.1997.5213

[14] Koshiol J , Wozniak A , Cook P , et al. Salmonella enterica serovar Typhi and gallbladder cancer: a case–control study and meta‐analysis. Cancer Med. 2016;5(11):3310‐3335. 27726295 10.1002/cam4.915 PMC5119987

[15] Pérez‐Moreno P , Riquelme I , García P , Brebi P , Roa JC . Environmental and lifestyle risk factors in the carcinogenesis of gallbladder cancer. J Pers Med. 2022;12(2):234. 35207722 10.3390/jpm12020234 PMC8877116

[16] Liu E , Sakoda LC , Gao YT , et al. Aspirin use and risk of biliary tract cancer: a population‐based study in Shanghai, China. Cancer Epidemiol Biomarkers Prev. 2005;14(5):1315‐1318. 15894693 10.1158/1055-9965.EPI-05-0032

[17] Prasai K , Tella SH , Yadav S , et al. Aspirin and statin use and the risk of gallbladder cancer. Cancers. 2021;13(5):1186. 33803387 10.3390/cancers13051186 PMC7967123

[18] Marcano‐Bonilla L , Schleck CD , Harmsen WS , et al. Aspirin, statins, non‐aspirin NSAIDs, metformin, and the risk of biliary cancer: a Swedish population‐based cohort study. Cancer Epidemiol Biomarkers Prev. 2022;31(4):804‐810. 35086822 10.1158/1055-9965.EPI-20-1322 PMC9136681

[19] Low EE , Demb J , Liu L , et al. Risk factors for early‐onset colorectal cancer. Gastroenterology. 2020;159(2):492‐501. 31926997 10.1053/j.gastro.2020.01.004 PMC7343609

[20] Bosetti C , Santucci C , Gallus S , Martinetti M , La Vecchia C . Aspirin and the risk of colorectal and other digestive tract cancers: an updated meta‐analysis through 2019. Ann Oncol. 2020;31(5):558‐568. 32272209 10.1016/j.annonc.2020.02.012

[21] Koshiol J , Bellolio E , Vivallo C , et al. Distribution of dysplasia and cancer in the gallbladder: an analysis from a high cancer‐risk population. Hum Pathol. 2018;82:87‐94. 30036595 10.1016/j.humpath.2018.07.015 PMC8579273

[22] Kim S . Ppcor: an R package for a fast calculation to semi‐partial correlation coefficients. Commun Stat Appl Methods. 2015;22(6):665‐674. 26688802 10.5351/CSAM.2015.22.6.665 PMC4681537

[23] R Core Team . R: a language and environment for statistical computing. R Foundation for Statistical Computing; 2022.

[24] Iyengar NM , Gucalp A , Dannenberg AJ , Hudis CA . Obesity and cancer mechanisms: tumor microenvironment and inflammation. J Clin Oncol. 2016;34(35):4270‐4276. 27903155 10.1200/JCO.2016.67.4283 PMC5562428

[25] Lammert F , Gurusamy K , Ko CW , et al. Gallstones. Nat Rev Dis Primers. 2016;2(1):16024. 27121416 10.1038/nrdp.2016.24

[26] Wang X , Chan AT , Slattery ML , et al. Influence of smoking, body mass index, and other factors on the preventive effect of nonsteroidal anti‐inflammatory drugs on colorectal cancer risk. Cancer Res. 2018;78(16):4790‐4799. 29921691 10.1158/0008-5472.CAN-18-0326 PMC6095723

[27] Koshiol J , Van De Wyngard V , McGee EE , et al. The Chile biliary longitudinal study: a gallstone cohort. Am J Epidemiol. 2021;190(2):196‐206. 33524121 10.1093/aje/kwaa199 PMC7850058

[28] Kim S , Keku TO , Martin C , et al. Circulating levels of inflammatory cytokines and risk of colorectal adenomas. Cancer Res. 2008;68(1):323‐328. 18172326 10.1158/0008-5472.CAN-07-2924 PMC2675825

[29] Baron JA , Cole BF , Sandler RS , et al. A randomized trial of aspirin to prevent colorectal adenomas. N Engl J Med. 2003;348(10):891‐899. 12621133 10.1056/NEJMoa021735

[30] Benamouzig R , Deyra J , Martin A , et al. Daily soluble aspirin and prevention of colorectal adenoma recurrence: one‐year results of the APACC trial. Gastroenterology. 2003;125(2):328‐336. 12891533 10.1016/s0016-5085(03)00887-4

[31] Chan AT , Giovannucci EL , Meyerhardt JA , Schernhammer ES , Curhan GC , Fuchs CS . Long‐term use of aspirin and nonsteroidal anti‐inflammatory drugs and risk of colorectal cancer. JAMA. 2005;294(8):914‐923. 16118381 10.1001/jama.294.8.914 PMC1550973

[32] Jackson SS , Van De Wyngard V , Pfeiffer RM , et al. Inflammatory profiles in Chilean Mapuche and non‐Mapuche women with gallstones at risk of developing gallbladder cancer. Sci Rep. 2021;11(1):3686. 33574564 10.1038/s41598-021-83300-2 PMC7878792

[33] Maker AV , Katabi N , Qin LX , et al. Cyst fluid interleukin‐1β (IL1β) levels predict the risk of carcinoma in Intraductal papillary mucinous neoplasms of the pancreas. Clin Cancer Res. 2011;17(6):1502‐1508. 21266527 10.1158/1078-0432.CCR-10-1561 PMC3065716

[34] Pu N , Chen Q , Zhang J , et al. Circulating cytokines allow for identification of malignant intraductal papillary mucinous neoplasms of the pancreas. Cancer Med. 2023;12(4):3919‐3930. 35871313 10.1002/cam4.5051 PMC9972143

[35] DelGiorno KE , Hall JC , Takeuchi KK , et al. Identification and manipulation of biliary metaplasia in pancreatic tumors. Gastroenterology. 2014;146(1):233‐244. 23999170 10.1053/j.gastro.2013.08.053 PMC3870045

[36] DelGiorno KE , Chung CY , Vavinskaya V , et al. Tuft cells inhibit pancreatic tumorigenesis in mice by producing prostaglandin D2. Gastroenterology. 2020;159(5):1866‐1881. 32717220 10.1053/j.gastro.2020.07.037 PMC7680354

[37] O'Leary CE , Sbierski‐Kind J , Kotas ME , et al. Bile acid–sensitive tuft cells regulate biliary neutrophil influx. Sci Immunol. 2022;7(69):1201. 10.1126/sciimmunol.abj1080 PMC9166270 35245089

