# Supplementary material for: Non‐steroidal anti‐inflammatory drug use and inflammatory markers associated with gallbladder dysplasia: A case–control analysis within a series of patients undergoing cholecystectomy
Source: Int J Cancer. 2024 Oct 31;156(7):1380–92. doi: 10.1002/ijc.35238 (PMC11789453; doi:10.1002/ijc.35238)
Supplement: Supplementary file 1 — Appendix S1. [file IJC-156-1380-s001.pdf]

Non-steroidal anti-inflammatory drug use and inflammatory markers associated with gallbladder dysplasia: a case-control analysis within a series of patients undergoing cholecystectomy

Lorena Rosa, Paz Cook, Ruth M Pfeiffer, Troy J Kemp, Allan Hildesheim, Burcin Pehlivanoglu, Volkan Adsay, Enrique Bellolio, Juan Carlos Araya, Ligia Pinto, Catterina Ferreccio, Gloria Aguayo, Eduardo Viñuela and Jill Koshiol

Table of contents

Supplementary Table 1. Percent of samples with detectable levels of inflammatory proteins and coefficients of variation (CVs) and intraclass correlation coefficients (ICCs).

Supplementary Table 2. Adjusted odds ratios (ORs) and 95% confidence intervals (95% CI) for the association of NSAIDs and dysplasia among normal weight, overweight and obese patients.

Supplementary Table 3. Statistically significant, strong correlations between inflammatory markers in controls and dysplasia cases.

Supplementary Table 4. Adjusted odds ratios (ORs), lower 95% confidence limit (LCL) and upper 95% confidence limit (UCL) for associations of inflammatory markers with dysplasia.

Supplementary Table 5. Comparison of characteristics of all CRS gallstones controls who currently use NSAIDs versus non- users.

Supplementary Table 6. Comparison of characteristics of all CRS dysplasia cases who currently use NSAIDs versus non- users.

Supplementary Figure 1. Schematic diagram of CRS patients that were analyzed to evaluate the associations of NSAID use and inflammatory proteins with dysplasia.

Supplementary Figure 2. Univariate associations of inflammatory markers with dysplasia and gallbladder cancer (GBC).

Supplementary Table 1. Percent of samples with detectable levels of inflammatory proteins and coefficients of variation (CVs) and intraclass correlation coefficients (ICCs).

Analyte	Total detectable (%)	CV	ICC	Reason if dropped from analysis
CCL19 (MIP-3B)	98.72	1.66	0.93	
CCL20 (MIP-3A)	84.62	14.54	0.76	
CXCL11 (I-TAC)	98.72	1.61	0.82	
CXCL6 (GCP2)	98.72	2.37	0.82	
CXCL9 (MIG)	98.72	0.59	0.73	
BCA-1	98.72	1.03	0.80	
CRP	100	5.91	0.97	
EGF	97.44	2.90	0.82	
ENA-78	98.72	0.38	0.90	
EOTAXIN	97.44	1.97	0.92	
EOTAXIN2	98.72	1.16	0.87	
FRACTALKINE	98.72	18.47	0.97	
GRO	97.44	0.68	0.89	
IL-16	82.05	28.82	0.77	
IL-33	25.64	7.57	0.77	
IL-12 p70	98.72	18.46	0.92	
IL-17A	98.72	22.16	0.89	
IL-1B	98.72	25.43	0.94	
IL-21	96.15	25.88	0.96	
IL-23	98.72	19.45	0.91	
IL-8	98.72	19.56	0.97	
IP-10	98.72	1.89	0.95	
MCP-1	97.44	1.37	0.82	
MCP-4	98.72	3.74	0.81	
MDC	97.44	1.96	0.96	
MIP-1B	97.44	8.85	0.77	
MIP-1D	98.72	0.73	0.89	
SAP	98.72	1.02	0.95	
sEGFR	98.72	1.01	0.73	
sTNFRI	98.72	3.08	0.75	
sTNFRII	98.72	0.44	0.98	
sVEGFR2	98.72	1.64	0.81	
sVEGFR3	98.72	1.76	0.94	

6CKINE	98.72	11.94	0.48	low ICC
CTACK	98.72	1.05	0.51	low ICC
FGF-2	91.03	63.35	0.98	high CV
G-CSF	52.56	43.81	0.98	high CV
GM-CSF	98.72	12.47	0.67	low ICC
IL-1RA	44.87	44.60	0.60	high CV & low ICC
IL-3	14.1	4.94	0.08	poor detectability & low ICC
IL-7	94.87	22.54	0.46	low ICC
IL-10	98.72	23.25	0.68	low ICC
IL-13	94.87	31.45	0.86	high CV
IL-4	98.72	30.36	0.74	high CV
IL-5	98.72	10.15	0.62	low ICC
IL-6	98.72	23.05	0.48	low ICC
IL-7	98.72	37.02	0.90	high CV
MCP-2	98.72	0.43	0.30	low ICC
MIP-1A	65.38	84.18	0.80	high CV
MIP-3A	98.72	6.79	0.67	low ICC
SAA	85.9	38.56	0.85	high CV
SCF	25.64	26.86	0.63	low ICC
SDF-1AB	96.15	8.29	0.41	low ICC
TARC	98.72	0.89	0.58	low ICC
TGF-a	89.74	42.29	0.95	high CV
TNF-b	12.82	27.41	0.96	poor detectability
TNF-a	97.44	7.93	0.01	low ICC
TPO	89.74	134.17	0.73	high CV
TRAIL	98.72	1.24	0.41	low ICC
TSLP	32.05	2.36	0.69	low ICC
IL-29 (IFNL-1)	76.92	36.98	0.98	high CV
sGP-130	100	0.96	0.60	low ICC
sIL-4R	53.85	99.16	0.88	high CV
sIL-6R	100	0.80	-0.01	low ICC
sILRII	98.72	115.53	0.46	high CV & low ICC

Supplementary Table 2. Adjusted odds ratios (ORs) and 95% confidence intervals (95% CI) for the association of NSAIDs and dysplasia among normal weight, overweight and obese patients.

Patients*	Current use of NSAIDs	Dysplasia cases, N (%)	Controls, N (%)	OR (95% CI)**	*P-value*†
Normal weight; N=374	No	22 (88.0)	229 (65.6)	Reference	-----
	Yes	3 (12.0)	120 (34.4)	0.30 (0.07-0.90)	0.013
	Total	25 (100.0)	349 (100.0)		
Overweight; N=800	No	29 (82.9)	533 (69.7)	Reference	-----
	Yes	6 (17.1)	232 (30.3)	0.49 (0.18-1.12)	0.079
	Total	35 (100.0)	765 (100.0)		
Obese; N=735	No	16 (76.2)	507 (71.0)	Reference	-----
	Yes	5 (23.8)	207 (29.0)	0.70 (0.23-1.84)	0.599
	Total	21 (100.0)	714 (100.0)		

*16 patients were excluded due to missing BMI data.
**Logistic regression models were adjusted for sex and categorical age.
†*P-value* was calculated by likelihood ratio test.

Supplementary Table 3. Statistically significant, strong correlations between inflammatory markers in controls and dysplasia cases.

Controls (N=136)				Dysplasia cases (N=68)			
**Variable 1**	**Variable 2**	**Coeff***	***P-value***	**Variable 1**	**Variable 2**	**Coeff***	***P-value***
**sTNFRI**	**sTNFRII**	**0.77**	**1.55E-25**	**IL-12p70**	**IL-1B**	**0.74**	**2.71E-11**
**FRACTALKINE**	**IL-1B**	**0.73**	**4.51E-21**	**IL-1B**	**IL-23**	**0.71**	**2.59E-10**
**IL-1B**	**IL-21**	**0.72**	**4.51E-21**	**IL-12p70**	**IL-17A**	**0.71**	**2.80E-10**
**IL-17A**	**IL-1B**	**0.69**	**2.90E-18**	**IL-1B**	**IL-21**	**0.70**	**6.14E-10**
**IL-1B**	**IL-23**	**0.68**	**1.16E-17**	**FRACTALKINE**	**IL-1B**	**0.67**	**5.68E-09**
**FRACTALKINE**	**IL-17A**	**0.66**	**1.08E-16**	**IL-12p70**	**IL-23**	**0.66**	**1.69E-08**
**IL-21**	**IL-23**	**0.66**	**4.01E-16**	**FRACTALKINE**	**IL-23**	**0.63**	**9.06E-08**
**IL-12p70**	**IL-21**	**0.64**	**2.06E-15**	**IL-12p70**	**IL-21**	**0.63**	**1.13E-07**
**IL-17A**	**IL-23**	**0.64**	**1.77E-15**	**FRACTALKINE**	**IL-12p70**	**0.62**	**2.15E-07**
**FRACTALKINE**	**IL-23**	**0.62**	**4.80E-14**	**EOTAXIN**	**MCP-1**	**0.61**	**3.74E-07**
**IL-12p70**	**IL-1B**	**0.62**	**4.76E-14**	**CRP**	**SAP**	**0.61**	**3.25E-07**
**IL-12p70**	**IL-17A**	**0.61**	**1.15E-13**	**CXCL9 (MIG)**	**IP-10**	**0.60**	**4.32E-07**
**IL-17A**	**IL-21**	**0.60**	**1.58E-13**	sEGFR	sVEGFR2	0.59	8.0E-07

*Adjusted Spearman's rank correlation coefficient by categorical age and sex. Strongly correlated markers (coeff≥0.6) are shown in bold and overlapped correlations between controls and dysplasia cases are highlighted in grey.

Supplementary Table 4. Adjusted odds ratios (ORs), lower 95% confidence limit (LCL) and upper 95% confidence limit (UCL) for associations of inflammatory markers with dysplasia.

	Univariate*				Multivariate**			
**Marker**	**OR**	**LCL**	**UCL**	***P-value***†	**OR**	**LCL**	**UCL**	***P-value***†
IL-33	0.30	0.12	0.77	0.012	0.47	0.16	1.34	0.160
BCA-1††	0.61	0.47	0.81	0.0005	0.74	0.54	1.01	0.056
GRO††	0.61	0.45	0.82	0.0009	0.64	0.45	0.91	0.014
CCL19 (MIP-3B)	0.70	0.53	0.92	0.011	0.89	0.64	1.23	0.480
sTNFRII	0.70	0.53	0.93	0.012	0.84	0.59	1.18	0.310
CXCL6 (GCP2)	0.71	0.53	0.94	0.015	1.05	0.74	1.49	0.780
CRP	0.73	0.55	0.96	0.027	0.95	0.68	1.34	0.780
MIP-1B	0.76	0.59	0.99	0.038	0.93	0.69	1.25	0.630
sVEGFR3	0.78	0.60	1.01	0.061	-----	-----	-----	-----
IL-21	0.82	0.63	1.06	0.13	-----	-----	-----	-----
IL-16	0.83	0.64	1.07	0.15	-----	-----	-----	-----
IL-12p70	0.83	0.64	1.08	0.17	-----	-----	-----	-----
sTNFRI	0.83	0.63	1.09	0.17	-----	-----	-----	-----
sVEGFR2	0.83	0.62	1.11	0.21	-----	-----	-----	-----
IP-10	0.84	0.64	1.09	0.19	-----	-----	-----	-----
MCP-4	0.84	0.65	1.09	0.19	-----	-----	-----	-----
IL-1B	0.85	0.66	1.08	0.18	-----	-----	-----	-----
IL-23	0.85	0.66	1.10	0.21	-----	-----	-----	-----
FRACTALKINE	0.85	0.65	1.11	0.24	-----	-----	-----	-----
MIP-1D	0.85	0.65	1.11	0.24	-----	-----	-----	-----
CCL20 (MIP-3A)	0.86	0.66	1.12	0.25	-----	-----	-----	-----
EGF	0.86	0.65	1.14	0.30	-----	-----	-----	-----
MCP-1	0.87	0.68	1.12	0.29	-----	-----	-----	-----
CXCL11 (I-TAC)	0.87	0.66	1.14	0.31	-----	-----	-----	-----
IL-17A	0.89	0.69	1.15	0.38	-----	-----	-----	-----
CXCL9 (MIG)	0.89	0.66	1.18	0.41	-----	-----	-----	-----
ENA-78	0.91	0.70	1.18	0.48	-----	-----	-----	-----

SAP	0.95	0.73	1.23	0.69	-----	-----	-----	-----
EOTAXIN	0.95	0.72	1.25	0.71	-----	-----	-----	-----
IL-8	0.96	0.75	1.22	0.74	-----	-----	-----	-----
sEGFR	1.05	0.80	1.38	0.71	-----	-----	-----	-----
MDC	1.07	0.82	1.38	0.63	-----	-----	-----	-----
EOTAXIN-2	1.28	0.98	1.67	0.07	-----	-----	-----	-----

*Conditional logistic regression models for each categorical marker adjusted by categorical age and sex.
**Conditional logistic regression models including markers with *P-value* < 0.05 adjusted by categorical age, sex, and current use of NSAIDs. †*P-value* for the trend across categories of each marker. ††Only BCA-1 and GRO passed Bonferroni correction (*P-value* < 0.0015). Multiple testing adjustment was not applied for our four *a priori*-identified inflammatory markers IL-16, CCL20, sTNFRI and CRP associated with increased risk of early-GBC (*P-value* ≤ 0.001)

Supplementary Table 5. Comparison of characteristics of all CRS gallstones controls who currently use NSAIDs versus non- users.

	**Current use of NSAIDs**		
	**No, N = 1,277**	**Yes, N = 565**	***P-value***
Sex, N (%)			0.083
Women	1013 (79.3)	427 (75.6)	
Men	264 (20.7)	138 (24.4)	
Categorical age, N (%)			0.026
<40 years	470 (36.8)	220 (38.9)	
40-50 years	323 (25.3)	116 (20.5)	
51-60 years	260 (20.4)	104 (18.4)	
>60 years	224 (17.5)	125 (22.1)	
Ethnicity, N (%)			0.6
Chilean/Latino	1209 (94.7)	533 (94.3)	
Mapuche	47 (3.7)	19 (3.4)	
N/A	21 (1.6)	13 (2.3)	
Education, N (%)			0.026
0-8 years	342 (26.8)	173 (30.6)	
9-12 years	666 (52.2)	261 (46.2)	
≥13 years	199 (15.6)	85 (15.0)	
N/A	70 (5.5)	46 (8.1)	
Categorical BMI, N (%)			0.2
Normal weight	229 (17.9)	120 (21.2)	
Overweight	533 (41.7)	232 (41.1)	

Obese	507 (39.7)	207 (36.6)	
N/A	8 (0.6)	6 (1.1)	
Consumption of alcohol in the last year, N (%)			0.06
No alcohol	790 (61.9)	378 (66.9)	
More than 1 days per month	486 (38.1)	187 (33.1)	
N/A	1 (0.1)	0 (0)	
Smoker habit, N (%)			0.7
Never smoker	1097 (85.9)	481 (85.1)	
Former smoker	68 (5.3)	36 (6.4)	
Current smoker	112 (8.8)	48 (8.5)	
Surgery type, N (%)			<0.001
Urgent	59 (4.6)	372 (65.8)	
Elective	1218 (95.4)	193 (34.2)	
Biliary cholic, N (%)			<0.001
No	237 (18.6)	21 (3.7)	
Yes	1008 (78.9)	538 (95.2)	
N/A	32 (2.5)	6 (1.1)	
Typhoid fever, N (%)			0.024
No	1206 (94.4)	533 (94.3)	
Yes	47 (3.7)	12 (2.1)	
N/A	24 (1.9)	20 (3.5)	
Chronic gastritis, N (%)			0.2
No	1210 (94.8)	527 (93.3)	
Yes	42 (3.3)	19 (3.4)	
N/A	25 (2.0)	19 (3.4)	

*Categorical comparisons were performed by Chi-square test for variables with n≥5 and fisher exact test for variables with n<5. N/A: not available. One participant was excluded from this analysis due to missing information of NSAID current use, giving a total of 1,842 CRS controls.

Supplementary Table 6. Comparison of characteristics of all CRS dysplasia cases who currently use NSAIDs versus non- users.

	**Current use of NSAIDs**		
	**No, N = 68**	**Yes, N = 14**	***P-value***
Sex, N (%)			0.495
Women	54 (79.4)	10 (71.4)	
Men	14 (20.6)	4 (28.6)	

Categorical age, N (%)			0.501
<40 years	15 (22.1)	6 (42.9)	
40-50 years	24 (35.3)	4 (28.6)	
51-60 years	15 (22.1)	2 (14.3)	
>60 years	14 (20.6)	2 (14.3)	
Ethnicity			1
Chilean/Latino	65 (95.6)	14 (100)	
Mapuche	2 (2.9)	0 (0)	
N/A	1 (1.5)	0 (0)	
Education, N (%)			0.248
0-8 years	20 (29.4)	2 (14.3)	
9-12 years	32 (47.1)	11 (78.6)	
≥13 years	9 (13.2)	1 (7.1)	
N/A	7 (10.3)	0 (0)	
Categorical BMI, N (%)			0.679
Normal weight	22 (32.4)	3 (21.4)	
Overweight	29 (42.6)	6 (42.9)	
Obese	16 (23.5)	5 (35.7)	
N/A	1 (1.5)	0 (0)	
Consumption of alcohol in the last year, N (%)			0.7
No alcohol	46 (67.6)	8 (57.1)	
More than 1 days per month	22 (32.4)	6 (42.9)	
Smoker habit, N (%)			0.342
Never smoker	63 (92.6)	12 (85.7)	
Former smoker	2 (2.9)	1 (7.1)	
Current smoker	3 (4.4)	1 (7.1)	
Surgery type, N (%)			0.009
Urgent	10 (14.7)	7 (50.0)	
Elective	58 (85.3)	7 (50.0)	
Biliary cholic, N (%)			1
No	2 (2.9)	0 (0)	
Yes	66 (97.1)	14 (100)	
Typhoid fever, N (%)			0.133
No	66 (97.1)	12 (85.7)	
Yes	1 (1.5)	2 (14.3)	
N/A	1 (1.5)	0 (0)	
Chronic gastritis, N (%)			0.534

No	65 (95.6)	13 (92.9)
Yes	2 (2.9)	1 (7.1)
N/A	1 (1.5)	0 (0)

*Categorical comparisons were performed by Chi-square test for variables with n≥5 and fisher exact test for variables with n<5. N/A: not available. Analysis was performed for dysplasia cases (N=82).

Supplementary Figure 1. Schematic diagram of CRS patients that were analyzed to evaluate the associations of NSAID use and inflammatory proteins with dysplasia.

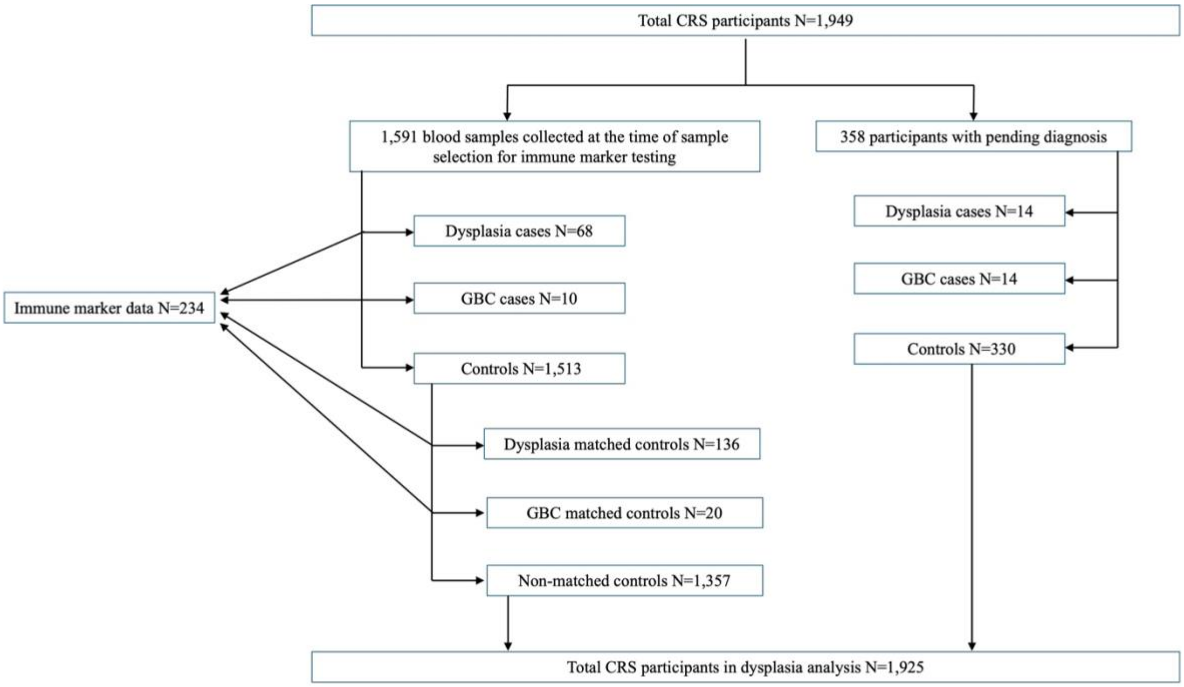

Supplementary Figure 2. Univariate associations of inflammatory markers with dysplasia and gallbladder cancer (GBC).

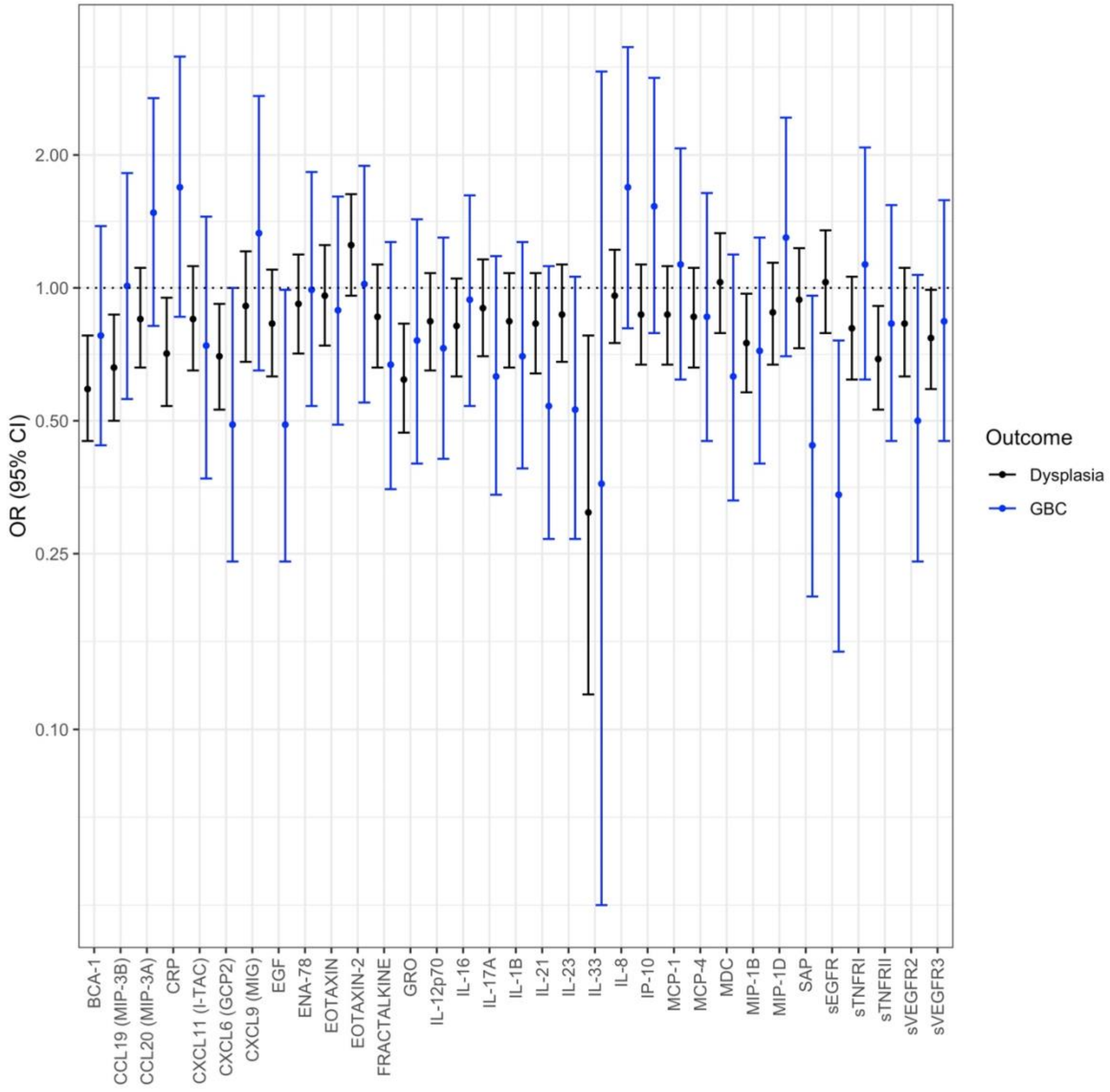

Odds ratios (ORs) and 95% confidence intervals (CIs) for each marker were calculated by polytomous logistic regression models adjusted by categorical age and sex. Univariate associations with dysplasia and GBC are indicated in black and blue; respectively.
